# Age and Alzheimer’s Disease-Related Oligodendrocyte Changes in Hippocampal Subregions

**DOI:** 10.3389/fncel.2022.847097

**Published:** 2022-04-07

**Authors:** Leah DeFlitch, Estibaliz Gonzalez-Fernandez, Ilan Crawley, Shin H. Kang

**Affiliations:** ^1^Biology Department, College of Science and Technology, Temple University, Philadelphia, PA, United States; ^2^Department of Neural Sciences, Lewis Katz School of Medicine, Temple University, Philadelphia, PA, United States; ^3^Shriners Hospitals Pediatric Research Center (Center for Neural Repair and Rehabilitation), Temple University, Philadelphia, PA, United States

**Keywords:** oligodendrocytes, hippocampus, myelination, OPC, age, Alzheimer’s disease

## Abstract

Oligodendrocytes (OLs) form myelin sheaths and provide metabolic support to axons in the CNS. Although most OLs develop during early postnatal life, OL generation continues in adulthood, and this late oligodendrogenesis may contribute to neuronal network plasticity in the adult brain. We used genetic tools for OL labeling and fate tracing of OL progenitors (OPCs), thereby determining OL population growth in hippocampal subregions with normal aging. OL numbers increased up to at least 1 year of age, but the rates and degrees of this OL change differed among hippocampal subregions. In particular, adult oligodendrogenesis was most prominent in the CA3 and CA4 subregions. In Alzheimer’s disease-like conditions, OL loss was also most severe in the CA3 and CA4 of APP/PS1 mice, although the disease did not impair the rate of OPC differentiation into OLs in those regions. Such region-specific, dynamic OL changes were not correlated with those of OPCs or astrocytes, or the regional distribution of Aβ deposits. Our findings suggest subregion-dependent mechanisms for myelin plasticity and disease-associated OL vulnerability in the adult hippocampus.

## Introduction

Oligodendrocytes (OLs) form myelin, lipid-rich insulation around axons, essential for saltatory axonal conduction in the CNS (Simons and Nave, 2015). OLs also support axons metabolically by providing glycolytic products, such as lactate and pyruvates, through monocarboxylate transporter 1 (MCT1) (Fünfschilling et al., 2012; Lee et al., 2012; Philips et al., 2021). Although a large proportion of OLs are generated early in postnatal development, OL generation continues into adulthood from abundant OL progenitor cells (OPCs) (Dawson et al., 2003; Rivers et al., 2008; Kang et al., 2010; Zhu et al., 2011; Young et al., 2013). Both oligodendrogenesis and myelination at later ages are dynamic processes, likely regulated by diverse mechanisms related to experience and neuronal activity (Gibson et al., 2014; Hughes et al., 2018; Mitew et al., 2018; de Faria et al., 2021). Interestingly, recent evidence suggests that juvenile or adult oligodendrogenesis and subsequent new myelination are required for complex motor learning (McKenzie et al., 2014), memory consolidation (Steadman et al., 2020), and the establishment of proper social behavior (Makinodan et al., 2012). Conversely, OL dysfunction and white matter abnormalities have been implicated in a growing number of psychiatric (Gibson et al., 2018) and neurodegenerative diseases, including Alzheimer’s disease (AD) (Dean et al., 2017; Nasrabady et al., 2018).

In recent years, multiple studies were performed to identify long-term, age-related changes in the OL population and myelination (Young et al., 2013; Yeung et al., 2014). However, current knowledge of adult OL changes is limited to selected brain areas, such as the corpus callosum and cerebral cortex, where OLs are either abundant (Young et al., 2013; Hill et al., 2018) or where longitudinal *in vivo* OL imaging is achievable (Hill et al., 2018; Hughes et al., 2018). In contrast, despite several previous studies, age-related changes in hippocampal OLs have not been clearly characterized. Most past studies relied on histological approaches with labeling of OLIG2 (Valério-Gomes et al., 2018), myelin proteins (Desai et al., 2009; Vanzulli et al., 2020) or both (Chao et al., 2020) to observe hippocampal OLs.

In this study, we used mouse tools for genetic labeling of OLs and OPC-specific Cre-loxP fate tracing to follow age-related oligodendroglial changes in the hippocampus. Our analysis focused on different hippocampal subregions, each of which may represent well-established neuronal circuits composed of specific axonal relays. Moreover, we determined how hippocampal OLs change in APP/PS1 mice, a mouse model of AD-like amyloidogenesis. Our results show that different hippocampal subregions exhibit distinct patterns of age-related OL growth and OL vulnerability to AD-like disease conditions. This suggests that hippocampal myelin plasticity underlies specific aspects of age- and AD-dependent cognitive changes related to hippocampal functioning.

## Results

### Oligodendrocyte-Specific EGFP Expression in *Mobp-EGFP* Mice

**FIGURE 1:**
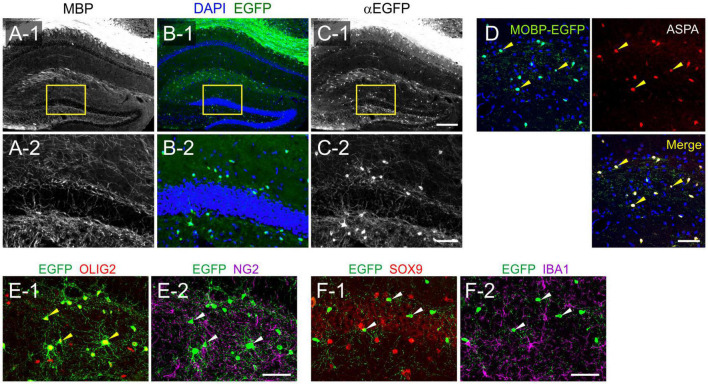
Genetic labeling of hippocampal oligodendrocytes with EGFP in *Mobp-EGFP* mice. **(A–C)** Fluorescence images of MBP **(A)**, naïve EGFP **(B)**, and immunostained EGFP **(C)**. **(A-2,B-2,C-2)** Correspond to the boxed areas of **(A-1,B-1,C-1)**, respectively. **(D)** Confocal images of EGFP and ASPA in the stratum lacunosum moleculare (SLM) of the CA1. **(E,F)** Confocal images of EGFP and cell-type-specific markers: OLIG2 **(E-1)** and NG2 **(E-2)** in the CA4 area, SOX9 **(F-1)** and IBA1 **(F-2)** in the stratum radiatum (SR) of CA1. Yellow arrowheads indicate co-localization of EGFP and immunolabeled marker proteins, while white arrowheads indicate EGFP non-overlapping with cell marker proteins. All images were obtained from a P30 Mobp-EGFP mouse. Scale bars: 200 μm **(A-1,B-1,C-1)**, 50 μm **(A-2,B-2,C-2,D)**, and 20 μm **(E,F)**.

**FIGURE 2:**
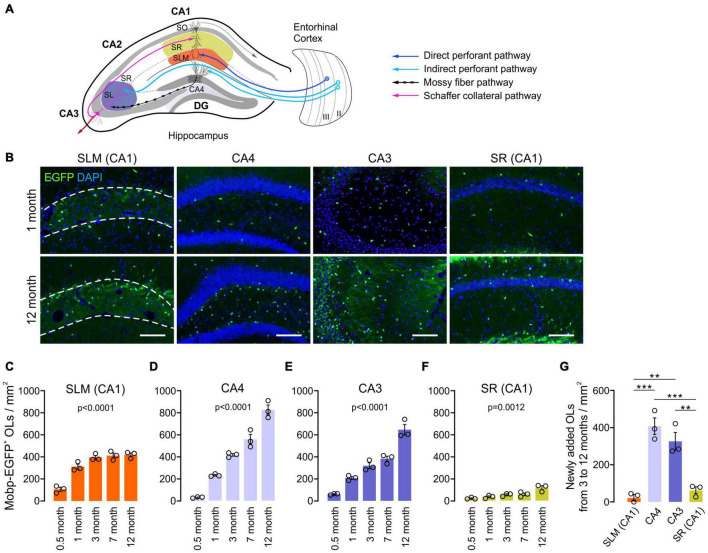
Oligodendrocyte density increases with age at different rates in different hippocampal regions. **(A)** Schematic diagram of the selected hippocampal subregions and related neuronal circuits. SO, stratum oriens; SR, stratum radiatum; SLM, stratum lacunosum moleculare; SL, stratum lucidum; DG, dentate gyrus. **(B)** Fluorescence images of EGFP^+^ OLs in 1- and 12-month-old *Mobp-EGFP* mice. The dashed lines indicate borders of the SLM. Scale bars: 100 μm. **(C–F)** Densities of EGFP^+^ OLs in the SLM of CA1 **(C)**, CA4, the hilus of the DG **(D)**, SR and SL of the CA3 **(E)**, and SR of the CA1 **(F)**. See Supplementary Table 1 for *p*-values of pairwise comparisons for **(C–F)**. **(G)** Change in EGFP^+^ OL density from 3 to 12 months of age. One-way ANOVA and Tukey’s test. **p < 0.01; ***p < 0.001. *n* = 3 mice for each group.

We first sought to understand how hippocampal OL densities change with age. For unambiguous OL identification and its quantification, we used *Mobp-EGFP* transgenic (Tg) mice, as done in previous studies (Kang et al., 2013; González-Fernández et al., 2018; Hughes et al., 2018). Whereas MBP immunostaining labels only OL processes (Figures 1A-1,A-2), naïve (Figures 1B-1,B-2) and immunostained (Figures 1C-1,C-2) EGFP signals preferably visualize OL soma in the hippocampus. All EGFP^+^ somas were co-labeled with anti-ASPA (a mature OL marker) (Figure 1D) and anti-OLIG2 (an OL lineage transcription factor) antibodies (Figure 1E-1). However, they were not co-localized with other cell-specific marker proteins, such as NG2 (an OPC marker) (Figure 1E-2), SOX9 (an astrocyte-specific transcription factor in the gray matter) (Sun et al., 2017; Figure 1F-1), or IBA1 (a microglia/macrophage marker) (Figure 1F-2). Notably, > 70% of OLIG2^+^ cells in the DG were NG2^+^ OPCs (at 1 month, data not shown), and a small fraction of the EGFP^+^ASPA^+^ cells were not OLIG2^+^ (at 12 months, data not shown). Thus, OLIG2 immunostaining, if used alone for OL quantification, requires caution in result interpretation (Valério-Gomes et al., 2018). Even with co-labeling of OLIG2 and myelin proteins (e.g., CNPase), OL numbers can be underestimated due to the ambiguity of myelin protein labeling and/or incomplete OL lineage detection with OLIG2. In contrast, EGFP in *Mobp-EGFP* mice specifically labels all OL somas, allowing for an accurate assessment of OL density in the hippocampus.

### Oligodendrocytes Increase With Age at Different Rates in Different Hippocampal Subregions

To understand how hippocampal OLs change with age, we examined hippocampi of *Mobp-EGFP* mice at the ages of 0.5, 1, 3, 7, and 12 months. Because oligodendrogenesis and new myelination are partly regulated by neuronal activity in the mature brain (Gibson et al., 2018; Mitew et al., 2018), we assumed that different neuronal circuits are subjected to differential use or patterning of neuronal activity with age. Therefore, we also asked whether hippocampal OL changes are subregion-dependent by analyzing four hippocampal subregions for OL changes: stratum lacunosum-moleculare (SLM) of CA1, the dentate gyrus (DG) hilus (hereafter called CA4), stratum lucidum (SL) and stratum radiatum (SR) of CA3, and SR of CA1 (Figure 2A). The density of EGFP^+^ OLs increased significantly with age in all examined hippocampal subregions, including adult age points (Figures 2B–F). However, the patterns of OL increase differed among those subregions. At P15, the SLM (CA1) exhibited the highest OL density compared to other regions (e.g., 106.5/mm^2^ in SLM vs. 28.8/mm^2^ in CA4, *p* = 0.03, Student’s *t*-test) (Figures 2C,D). Except for the SR of CA1, all areas showed increased OL density, particularly from P15 to 1 month of age (Figures 2B–F). At later ages, however, only CA4 and CA3 regions showed robust and continuous adult OL accumulation (Figures 2C–G and Supplementary Table 1). In contrast, we observed only an insignificant increase in adult OLs in the SLM and a stagnant adult OL number in the SR of CA1 (Figures 2C–G and Supplementary Table 1). These results indicate subregion-specific mechanisms that differentially drive adult OL generation in the hippocampus.

### Fate Analysis of Adult Oligodendrocytes Progenitor Cells in the Hippocampus

**FIGURE 3:**
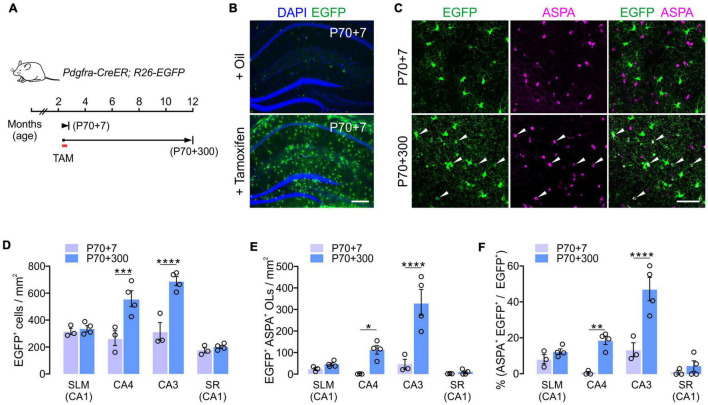
Cre-loxP fate analysis of adult OPCs in the hippocampus. **(A)** Experimental scheme of tamoxifen administration and sampling of *Pdgfra-CreER; R26-EGFP* mice. **(B)** Tamoxifen injections into P70 mice led to the EGFP expression in most hippocampal OPCs 7 days later (P70 + 7). **(C)** Confocal images of EGFP^+^ cells in CA3 at P70 + 7 or P70 + 300. Scale bars: 200 μm **(B)** and 50 μm **(C)**. **(D,E)** Densities of EGFP^+^ cells **(D)** and EGFP^+^ ASPA^+^ OLs **(E)**. **(F)** Percentages of ASPA^+^ OLs among EGFP^+^ cells. Two-Way ANOVA and Sidak’s multiple comparison test. *p < 0.05; **p < 0.01; ***p < 0.001; ****p < 0.0001. *n* = 3 or 4 mice for each group.

To confirm the subregion specific OL changes in the hippocampus, we followed the fate of adult OPCs in the aging brain. To analyze OPC fates, we crossed *Pdgfra-CreER* mice, a line of OPC-specific tamoxifen-inducible Cre mice (Kang et al., 2010) with *R26-EGFP* (RCE) Cre reporter mice (Figure 3A). Multiple injections of tamoxifen into *Pdgfra-CreER; RCE* mice between P70 and P73 resulted in EGFP expression in > 65% of NG2^+^ OPCs in CA1 and CA4 of the hippocampus when the brains were observed at P77 (P70 + 7) (Figure 3B). At P70 + 7, only 2 and 12% of EGFP^+^ cells were ASPA^+^ OLs in CA4 and CA3, respectively (Figures 3C,F). To estimate new oligodendrogenesis after P70 for the following 10 months, 12-month-old *Pdgfra-CreER; RCE* mice were examined. Consistent with the results of EGFP^+^ OL quantification (Figure 2), 12-month-old mice had much higher densities of EGFP^+^ cells and EGFP^+^ ASPA^+^ mature OLs in the CA4 and CA3, compared to younger counterparts (Figures 3C–E). 12-month-old mice also had a greater proportion of mature OLs among EGFP^+^ cells in the CA3 and CA4 region, but not in the SLM or SR of CA1 (Figure 3F), suggesting a greater accumulation of EGFP^+^ ASPA^+^ OLs in CA3 and CA4 with age. Thus, both our quantitative analysis of MOBP-EGFP^+^ OLs and adult Cre-loxP OPC fate analysis suggest that the CA3 and CA4 region are the most active areas of adult oligodendrogenesis in the hippocampus. These findings suggest that subsets of hippocampal neurons are subjected to new adult myelination at different rates.

### Age-Related Changes in Myelinated Processes in the Hippocampus

**FIGURE 4:**
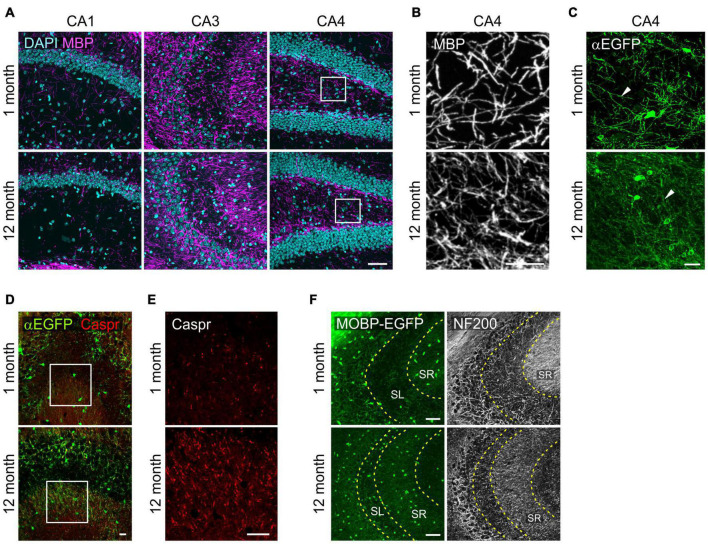
Changes in oligodendrocyte processes in the hippocampus with age. **(A)** Confocal images of MBP in the SR1 of CA1, CA3, and CA4. **(B)** The boxed areas of CA4 in **(A)** are magnified. **(C)** Confocal images of EGFP^+^ OL processes in the CA4. **(D)** Confocal images of EGFP^+^ OLs and Caspr in CA3. **(E)** Images of Caspr in the boxed areas in **(D)** are magnified. **(F)** Confocal images of MOBP-EGFP and NF200 in CA3. Scale bars: 50 μm **(A,F)** and 20 μm **(B–E)**.

With confocal microscopy of MBP, we noticed that MBP^+^ processes in 12-month-old-mice were thinner or shorter than those of 1-month-old mice (Figures 4A,B). These differences suggest more compacted myelin status and shorter internodes at old age as shown before (Young et al., 2013). Similar changes were observed more clearly with EGFP-stained OL processes in *Mobp-EGFP* mice (Figure 4C). The arborization patterns of EGFP^+^ OL processes in CA4 were more complex, but processes were thinner, at 12-months than at 1-month (Figure 4C). Of note, the differential increases of OL densities in hippocampal subregions (CA3 and CA4) were not correlated to changes in MBP immunoreactivity (pixel density) in the corresponding areas (data not shown). The disproportionate change in MBP^+^ immunoreactivities relative to OL number may be partly related to poor access of MBP (or GFP) antibodies to the compact myelin in the aged CNS (Gonsalvez et al., 2019).

However, we observed marked increases in Caspr^+^ puncta, a paranodal marker, in the SR of CA3 from 1 to 12 months with age, indicating increases in nodes of Ranvier as can be predicted by OL density increases (Young et al., 2013) in this hippocampal subregion (Figures 4D,E). To assess a possible change in axonal density in the SR of CA3 with age, we used anti-NF200 immunostaining as a neurofilament marker. NF200^+^ immunoreactivity changed from a fibrous to a more granulated pattern with age (Figure 4F), and its density was significantly reduced (160.5 ± 8.8 for 1 month vs. 71.8 ± 2.3 for 12 months; *p* < 0.005). We interpreted these changes as reflecting either axonal property changes or reduced density in this subregion with age. These results suggest that OL increases in CA3 are not driven by NF200^+^ axon increases with age.

### Oligodendrocytes Progenitor Cell and Astrocyte Number Change With Age in the Hippocampus

**FIGURE 5:**
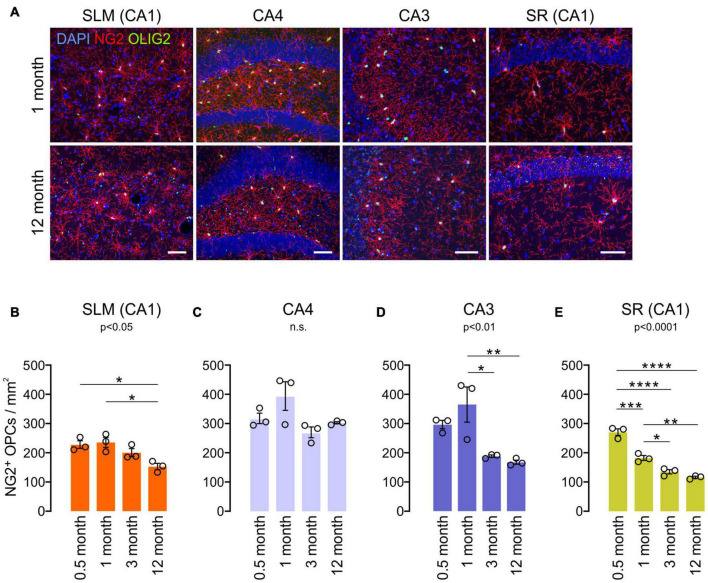
Age-related changes in NG2^+^ OPC density in the hippocampus. **(A)** Confocal images of NG2 and OLIG2 in 1- and 12-month-old mice. Scale bars: 50 μm. **(B–E)** OPC densities in the SLM of the CA1 **(B)**, CA4 **(C)**, CA3 **(D)**, and SR of the CA1 **(E)**. *p < 0.05; **p < 0.01; ***p < 0.001; ****p < 0.0001. One-way ANOVA and Tukey’s multiple comparisons test. *n* = 3 mice for each group.

To understand other glial changes with age in the hippocampus, OPC changes were analyzed in the same four hippocampal subregions at four different ages (0.5, 1, 3, and 12 months). OPCs are a major population of proliferating cells, and their density is known to be maintained in the adult brain (Dawson et al., 2003). Our results showed that NG2^+^ OPC densities decreased from 1 to 12 months of age in most subregions (Figures 5A–E), but in CA4, such an OPC decrease was not observed (Figure 5C). In our analysis, however, different hippocampal subregions displayed differing rates of decline throughout the lifespan. For example, from 1 to 3 months of age, there was a significant decline in OPC number in the CA3 and SR of CA1, while the SLM of CA1 and CA4 do not exhibit such an early decline (Figures 5B–E). From 3 months throughout adulthood (to 12 months of age), there was virtually no significant change in OPC number in all regions (Figures 5B–E). Thus, our results indicate that hippocampal OPC number is relatively stable in adulthood after initial decreases before 3 months, consistent with previous results obtained from other CNS areas (Zhu et al., 2011). Moreover, these age-related OPC changes in adulthood did not inversely correlate with concurrent OL increases in each subregion (Figures 2C–G).

**FIGURE 6:**
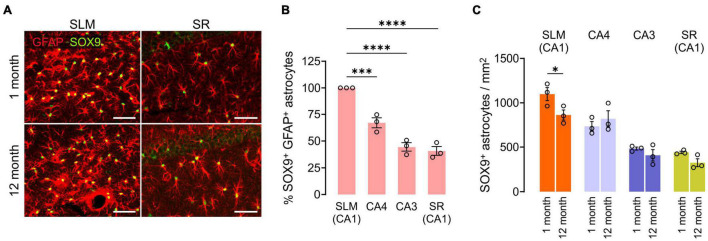
Changes in hippocampal astrocyte number with age. **(A)** Fluorescence images of GFAP and SOX9 in the SLM (left) and SR (right) of CA1. Scale bars: 50 μm. **(B)** Relative number of SOX9^+^ GFAP^+^ astrocytes in hippocampus subregions at 1 month. One-Way ANOVA and Dunnett’s multiple comparison test. ***p < 0.001; ****p < 0.0001. **(C)** Astrocyte density change from 1 to 12 months of age. Two-Way ANOVA and Sidak’s multiple comparison test. *p < 0.05. *n* = 3 mice for each group.

We also compared GFAP^+^SOX9^+^ hippocampal astrocyte densities at 1 and 12 months of age. Except for the subgranular zone (SGZ) of the DG (Sun et al., 2017), anti-SOX9 immunoreactivities were localized in the nuclei of GFAP^+^ astrocytes (Figure 6A). We noted that there were prominent regional differences in densities of SOX9^+^ GFAP^+^ astrocytes in the hippocampus at 1 month, and astrocytes were usually at higher density in SLM and CA4 than in CA3 and SR of the CA1 at both 1 and 12 months of age (e.g., 1098.6/mm^2^ in SLM vs. 441.3 in the SR of CA1 at 1 month, *p* < 0.0001, One-way ANOVA) (Figures 6A,B). However, unlike OLs and OPCs, SOX9^+^ cell density did not decrease significantly with age in most subregions except for the SLM of CA1 (Figure 6C). The results thus far suggest that each of three macroglial populations (i.e., OLs, OPCs, and astrocytes) follows a distinct regional pattern for its age-related changes in the hippocampus.

### Prominent Oligodendrocyte Loss in CA3 and CA4 of the Hippocampus in APP/PS1 Mice

**FIGURE 7:**
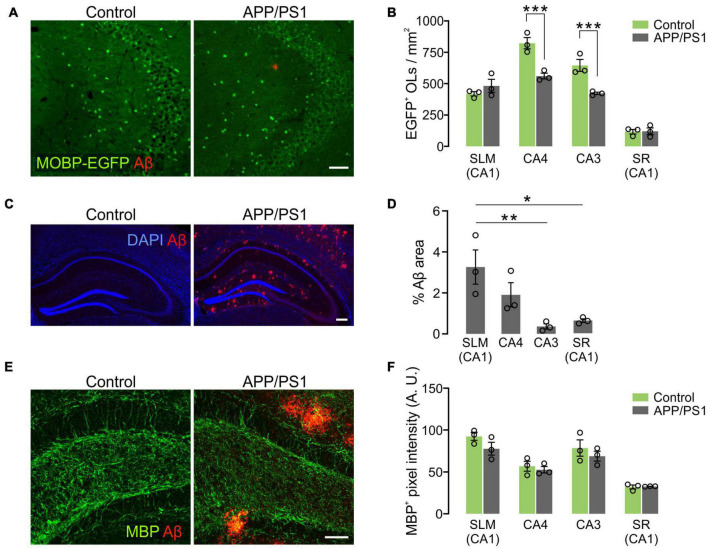
Loss of hippocampal oligodendrocytes in 12-month-old APP/PS1 mice. **(A)** Fluorescence images of EGFP^+^ OLs and Aβ in the CA3 of Mobp-EGFP; ± APP/PS1 mice. **(B)** Quantification of EGFP^+^ OL density. **(C)** Confocal images of MBP and Aβ in CA4. Optical thickness: 7 μm. **(C)** Fluorescence images showing distribution of Aβ deposits in the hippocampus of 12-month-old APP/PS1 mice. **(D)** Percentage of Aβ plaque areas. **(E)** Confocal images of MBP and Aβ in CA4. Optical thickness: 7 μm. **(F)** Quantification of MBP^+^ pixel values. Two-Way ANOVA and Sidak’s multiple comparisons test **(B,F)**. One-Way ANOVA and Tukey’s test **(D)**. *p < 0.05; **p < 0.01; ***p < 0.001. *n* = 3 mice for each group. Scale bars: 50 μm **(A,E)** and 200 μm **(C)**.

To understand how OLs are affected by AD-like disease conditions in different hippocampal subregions, OL densities were compared between 12-month-old control and APP/PS1 mice. Whereas OL densities of the two groups were comparable in the SLM and SR of CA1, there was a significant decrease in EGFP^+^ OLs in CA3 and CA4 of APP/PS1 mice (Figures 7A,B). Interestingly, the area of Aβ plaques in CA3 was smaller than those in other hippocampal subregions in APP/PS1 mice (Figures 7C,D). Moreover, there was no loss of EGFP^+^ OLs in the SLM of CA1 (Figure 7B), although this region had the highest levels of Aβ plaques among the examined areas in 12-month-old APP/PS1 mice (Figures 7C,D). Therefore, the regional pattern of hippocampal OL loss in APP/PS1 mice does not appear to be correlated with the levels of the extracellular Aβ plaque deposits. However, densitometric analysis of MBP^+^ immunoreactivities did not show MBP reduction as clearly as the loss of MOBP-EGFP^+^ OLs in APP/PS1 mice (Figures 7E,F). These results suggest that AD-like disease conditions cause hippocampal OL loss in a subregion-dependent manner, but Aβ plaque-induced cell toxicity is not a primary cause of the OL loss in 12-month-old APP/PS1 mice. Our results also raise an issue that histological analysis of MBP-immunoreactivities may not always reliably reflect pathological OL loss as noted by others (Gonsalvez et al., 2019).

### Hippocampal Adult Oligodendrogenesis Is Not Impaired in APP/PS1 Mice

**FIGURE 8:**
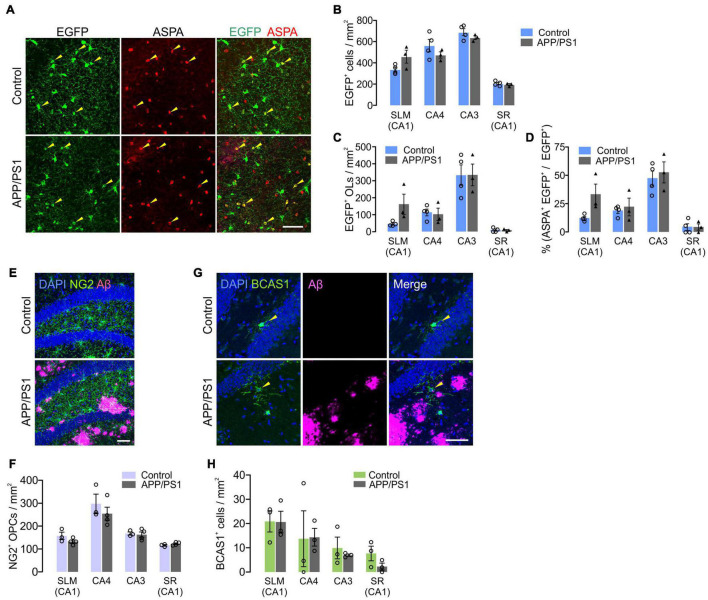
Adult oligodendrogenesis from hippocampal OPCs in APP/PS1 mice. **(A)** Confocal images of EGFP and ASPA in CA3 of the hippocampus in 12-month-old *Pdgfra-CreER; R26-EGFP;* ± *APP/PS1* (P70 + 300) mice. **(B,C)** Densities of EGFP^+^ cells **(B)** and EGFP^+^ ASPA^+^ OLs **(C)**. **(D)** Percentage of ASPA^+^ OLs among EGFP^+^ cells. **(E)** Confocal images of NG2^+^ OPCs and Aβ deposits in CA4 of 12-month-old control and APP/PS1 mice. **(F)** Quantification of OPCs. **(G)** Confocal images of BCAS1^+^ newly formed myelinating OLs and Aβ deposits in CA4. **(H)** Quantification of BCAS1^+^ cells. Scale bars: 50 μm **(A,E,G)**. No significant change was observed between control and APP/PS1 mice. Two-Way ANOVA and Sidak’s multiple comparisons test. *n* = 3 or 4 mice for each group.

It is possible that the subregion specific OL loss in APP/PS1 mice was due to decreased adult oligodendrogenesis. To test this possibility, we crossed *Pdgfra-CreER; RCE* with *APP/PS1* mice and administered tamoxifen into *Pdgfra-CreER; RCE;* ± *APP/PS1* mice at P70. The fates of P70 OPCs were analyzed by examining EGFP^+^ cells in the hippocampus at 12 months (P70 + 300). Although there was OL loss in CA3 and CA4 of *APP/PS1* mice (Figure 7B), we found no significant difference between control and *APP/PS1* groups in the densities of total EGFP^+^ cells (Figures 8A,B), EGFP^+^ ASPA^+^ OLs (Figures 8A,C), or the percentage of mature OLs among EGFP^+^ cells (Figure 8D) in all hippocampal subregions. The NG2^+^ OPC densities were also comparable between control and APP/PS1 mice (Figures 8E,F). We also quantified newly formed myelinating OLs that are marked with anti-breast carcinoma amplified sequence 1 (BCAS1) antibodies (Fard et al., 2017), but observed very few BCAS1^+^ cells in the hippocampus, with no difference in their numbers between the two groups (Figures 8G,H). These results suggest that the selective loss of CA3 and CA4 OLs in *APP/PS1* mice is not attributed to impairment in adult oligodendrogenesis or loss of OPCs. However, despite the intact potential of OPCs to differentiate into mature OLs, adult oligodendrogenesis may not be sufficient to restore normal OL density in the CA3 and CA4 of APP/PS1 mice.

**FIGURE 9:**
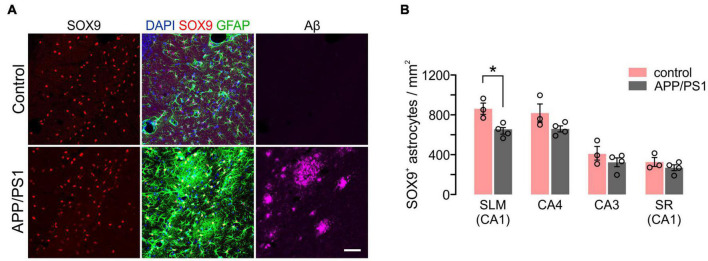
Density of hippocampal astrocytes in 12-month-old control and APP/PS1 mice. **(A)** Confocal images of Sox9^+^ GFAP^+^ astrocytes and Aβ in SLM. Scale bars: 50 μm. **(B)** Quantification of astrocytes. Two-Way ANOVA and Sidak’s multiple comparisons test. *p < 0.05. *n* = 3 or 4 mice for each group.

We also analyzed disease-related changes in hippocampal astrocyte density in APP/PS1 mice. Notably, despite marked upregulation of GFAP near Aβ deposits (Figure 9A), the number of SOX9^+^ astrocytes were not increased in all hippocampal regions (Figure 9B). Instead, SOX9^+^ cell densities were significantly decreased in the SLM of 12-month-old APP/PS1 mice (Figure 9B), which indicates a different regional pattern than that of OL loss in these mice (Figure 7B). These results suggest that distinct mechanisms underlie glial cell loss for OLs and astrocytes in APP/PS1 mice.

In summary, we found that CA3 and CA4 are the areas of most active adult oligodendrogenesis in the hippocampus, and that, in the same areas, OLs are more susceptible to diseased conditions in APP/PS1 mice. In contrast, astrocytes in SLM of the CA1, but not in other regions, undergo age or disease-related loss.

## Discussion

### Adult Oligodendrocyte Density Changes With Age

The thickness of white matter continues to increase in the adult brain to certain mid-ages in humans (Tamnes et al., 2010; Westlye et al., 2010; Lebel et al., 2012). However, to what degree new OL addition contributes to white matter change is unclear (Walhovd et al., 2014; Lebel and Deoni, 2018). OL numbers remain stable in the human corpus callosum from childhood into late ages, with no overt signs of OL turnover, although the myelin exchange rate is high (Yeung et al., 2014). In contrast, new OLs are continuously added to the upper layers of the mouse cortex (Hill et al., 2018; Hughes et al., 2018), even up to 2 years of age (Hill et al., 2018). Genetic lineage tracing of OPCs also reveals continuing oligodendrogenesis in the adult mouse corpus callosum, although the rate of oligodendrogenesis declines with age (Rivers et al., 2008; Kang et al., 2010; Young et al., 2013). Thus, OLs increase with age in at least some brain areas— however, the rate and pattern of age-dependent oligodendroglial growth likely differ depending on species, the brain region, and the time window of OL assessment.

### Hippocampal Oligodendrocytes and Myelinated Processes in Normal Aging

In this study, we found an age-dependent OL increase in the mouse hippocampus up to 12 months of age, and our results reveal that different hippocampal subregions exhibit different rates of OL number growth, indicating subregion-specific mechanisms for late myelination in this brain area. The hippocampus is the central brain area for learning and memory formation (Moser et al., 2015; Eichenbaum, 2017) and is an OL-sparse gray matter area in mice. The importance of axonal myelination in the region has not been clearly defined (Bonetto et al., 2021). Nonetheless, several studies noted age-related decline in CNPase^+^ fibers in CA1 (from 4 to 14 months) (Hayakawa et al., 2007), or MBP^+^ processes in CA3 (from 6 to 24 months) (Vanzulli et al., 2020), emphasizing myelin loss in the aged hippocampus. However, interpreting CNP or MBP immunostaining results requires caution, because tissue penetration of antibodies against myelin proteins to aged myelin is often limited by its lipid-rich compact myelin status (Gonsalvez et al., 2019). Our confocal microscopy of MBP^+^ or EGFP^+^ cell processes showed increased and more complex patterns of OL processes in 12-month-old brains than in young counterparts, although OL process or internodes appeared much thinner in older mice. We interpret these observations as evidence that the number of OL processes and myelin internodes increase in parallel with OL soma in this age window.

### Quantitative Analysis of Oligodendrocytes

Instead of assessing myelin protein antigenicity, we directly quantified discrete EGFP-labeled OL soma and profiled age-dependent OL addition in the hippocampus. Our results reveal that hippocampal subregions CA3 and CA4 are the most active and steady areas of adult oligodendrogenesis. These results were further supported by genetic lineage tracing results of OPCs that were EGFP-labeled after P70. Compared to the CA3 and CA4, new OL addition to the CA1 was insignificant during the same age window. The SLM of CA1, where entorhinal cortex layer III neurons project their axons, does not exhibit further OL addition from 3 months, despite its abundance of OLs relative to other hippocampal subregions. In earlier studies, similar myelin increases in CA3 and CA4 regions of the adult brain have not been noted. Interestingly, Abrahám et al. (2010) observed that, unlike other hippocampal subregions, the levels of MBP^+^ myelinated axons in the CA4 further increased from 11 years of age to adulthood in humans. Although this study did not assess myelin changes after adulthood, their results suggest late or continuous myelination in CA4.

### Oligodendroglial Changes Are Distinct From Those of Oligodendrocytes Progenitor Cells and Astrocytes

In contrast to OLs’ steady increase in CA3 and CA4, OPC densities decline until 3 months of age but either remain unaltered or decrease to a small degree afterward. Thus, the prominent OL increases in CA3 and CA4 are not inversely correlated with a change in the number of OPCs; instead, they may be caused by other extracellular mechanisms that stimulate the OPC-to-OL transition. Our OPC quantification results are different from other data suggesting that OPC density decreases with age in the hippocampus (Chacon-De-La-Rocha et al., 2020; Vanzulli et al., 2020). However, the discrepancy among studies may be due to different ages examined in previous studies (14 or 24 months as an old age point). In contrast, we quantified OPC numbers only up to 12 months of age.

Astrocytes display more reactive transcriptomal changes with age in the cortex, hippocampus, and straitum (Clarke et al., 2018). Nonetheless, astrocyte densities appear to be stable throughout life on the basis of the lack of changes in S100β^+^ cell density in the cortex and CA3 of the hippocampus (Grosche et al., 2013), or single cell-transcriptome data obtained from the whole mouse brain (Ximerakis et al., 2019). Consistent with these observations, our quantification of GFAP^+^ Sox9^+^ cells suggests that astrocytes in most hippocampal subregions are stable with age. One exception is the SLM of CA1, where the astrocyte density is the highest among hippocampal subregions. The significant astrocyte decrease in the SLM with age points to a regional pattern in astroglial changes different than that of OLs. Thus, all three macroglial cell populations in the hippocampus may follow distinct patterns of number change with age. Although astrocytes and microglia play critical roles in the early OL development and survival throughout the CNS (Domingues et al., 2016), OL density changes in local brain regions at later ages may be subjected to additional regulatory mechanisms, including circuit-specific neuronal activities (Kougioumtzidou et al., 2017; Hughes and Stockton, 2021). It is unclear whether neuronal activity controls astrocyte density.

### Subregion-Specific Oligodendroglial Susceptibility to Alzheimer’s Disease-Like Conditions

Oligodendroglial abnormalities have been implicated in various age-related neurodegenerative diseases. In particular, abnormal changes in OL-lineage cells and myelin damages were observed in postmortem AD brain tissues (Mitew et al., 2010; Behrendt et al., 2013; Tse et al., 2018) and in mouse models of AD (Desai et al., 2009; Tse et al., 2018; Chacon-De-La-Rocha et al., 2020; Vanzulli et al., 2020). For example, in 3xTg-AD mice, significant losses of MBP^+^ immunoreactivities were observed in CA1 (Desai et al., 2009) at different ages. Subtle changes in OPC morphology or density have also been noted in mouse models of AD (Chacon-De-La-Rocha et al., 2020; Vanzulli et al., 2020) and patients (Nielsen et al., 2013; Zhang et al., 2019), although whether morphological changes in OPCs are functionally linked to OL change is unclear. A recent study indirectly assessed OL loss by measuring the decrease in fractions of CNPase^+^ cells among Olig2^+^ cells in the CA1, CA2-3, and DG of 10-month-old APP/PS1 mice (Chao et al., 2020). We also observed a loss of hippocampal OLs in the same mouse model, but when observed at 12 months, OL loss was regionally restricted to CA3 and CA4. It is not clear why the results differed between these two studies. Nonetheless, it should be noted that different methods for OL identification may have different sensitivity for OL detection.

We found that the regional patterns of OL loss in the hippocampus are not inversely correlated with the levels of Aβ deposits in 12-month-old APP/PS1 mice, suggesting another mechanism driving OL loss at this stage of the disease. Indeed, OL loss precedes Aβ pathology in another AD mouse model (Tse et al., 2018). Moreover, Cre-loxP-dependent OPC fate analysis results indicate that OPC’s potential for new oligodendrogenesis was not impaired in the CA3 and CA4. Therefore, our results suggest that the OL loss in CA3 and CA4 in APP/PS1 mice may be due to the death of early-born OLs without sufficient OL replenishment, despite intact OL generation. Unlike our findings, recent studies reported increased OL turnover in the diseased hippocampus. Those conclusions were based on the observed increase of new adult born OLs without net OL density change in another AD mouse model (J20 or PDGF-APP_*Sw*,*Ind*_) (Ferreira et al., 2020) and a mouse model of tauopathy (MAPT ^*P*301S^) (Ferreira et al., 2020, 2021).

Changes in hippocampal astrocytes have been observed in mouse models of AD, including reduced astrocyte number (Olabarria et al., 2011; Beauquis et al., 2013) and atrophy or hypertrophy of astrocytes, dependent on their association with Aβ deposits (Rodríguez et al., 2009; Olabarria et al., 2010). We also identified a decrease in astrocyte density that was specific to the SLM of CA1. Our findings suggest that the OLs and astrocytes are subjected to different AD-related stresses which are subregion-specific.

### Significance of Continuous Oligodendroglial Addition to CA3 and CA4 With Age

It is unclear why CA3 and CA4 in the hippocampus are particularly active in adult oligodendrogenesis, but also vulnerable to AD-like conditions in APP/PS1 mice. The significance of the hippocampal subregion-dependent differential myelination will also be an important question. DG and CA3 are known to be critical for pattern separation, the formation of distinct memories from experiences with overlapping elements (Leutgeb et al., 2007; Bakker et al., 2008; Duncan and Schlichting, 2018). With age, there can be region-specific changes to the DG and CA3 activity, associated with deficits to pattern separation (Yassa et al., 2011a,b; Reagh et al., 2018). Thus, active adult myelination in these regions may support such memory distinctions, and its loss may lead to confused memory.

Collectively, our results reveal subregion-specific and dynamic adult OL changes in the hippocampus through healthy aging or in AD-like disease conditions. These findings warrant further studies to identify specific subgroups of hippocampal neurons as the targets of adult myelination and determine how this region-specific myelination shapes memory processes and other aspects of cognition.

## Materials and Methods

### Mice

*Pdgfra-CreER™* (RRID:IMSR_JAX:018280) and *Mobp*-*EGFP* (developed by GENSAT, MMRRC stock #030483-UCD, RRID:MMRRC_030483-UCD) mice were described previously (Kang et al., 2013), and are available from the Jackson Laboratory and the Mutant Mouse Resource & Research Centers (MMRRC), respectively. *R26-EGFP* mice (RCE) (Sousa et al., 2009) (MMRRC stock #032037-JAX, RRID:MMRRC_032037-JAX) were developed by Dr. Gordon Fishell’s laboratory (Harvard University) and indirectly obtained from Dr. Bergles (Johns Hopkins University). *APP/PS1* (APPswe, *PSEN1*ΔE9; MMRRC stock #034829-JAX, RRID:MMRRC_034829-JAX) (Jankowsky et al., 2001) mice were obtained from MMRRC. Mice of both genders were used in an unbiased manner and had mixed genomic backgrounds of B6SJL, CH3, C57BL/6, and 129. All experiments were carried out in compliance with the animal protocols approved by the Institutional Animal Care and Committee (IACUC) at Temple University.

### Tamoxifen Administration

Cre activity was induced with tamoxifen (Sigma-Aldrich, Cat# T5648) administration to *Pdgfra-CreER* mice. Tamoxifen was dissolved (20 mg/ml) in a mixture of sunflower seed oil-ethanol (10:1), and then ethanol was evaporated in a vacuum concentrator for 30 min. Forty mg/kg (b.w.) of tamoxifen was intraperitoneally (i.p.) injected twice daily with at least a 6-h interval between injections. A total of 10 doses of tamoxifen was injected into the *Pdgfra-CreER mice; R26-EGFP;* ± *APP/PS1* mice between P70 and P74.

### Tissue Preparation

Mice were deeply anesthetized with sodium pentobarbital (70 mg/kg, i.p.) and subjected to brief trans-cardiac perfusion with PBS and subsequently 4% paraformaldehyde (PFA, in 0.1 M phosphate buffer, pH 7.4). Brains were isolated and incubated in 4% PFA at 4°C overnight for post-fixation. Fixed brains were then incubated in 30% sucrose (in PBS) at 4°C for at least 36 h for cryoprotection. Tissues were embedded and frozen on Tissue-Tek optimum cutting temperature (O.C.T.) compound with dry ice. Twenty or 35 μm-thick coronally-cut brain sections were prepared using a cryostat (Leica) and kept in PBS complemented with 0.1% sodium azide.

### Immunofluorescence

Four to 5 brain sections per mouse were used for immunofluorescence and image analysis. After brain sections were rinsed in PBS (for 5 min, three times), they were permeabilized with 0.3% Triton X-100 (in PBS) for 5 min and then incubated in a blocking solution (0.3% Triton X-100, 5% normal donkey serum in PBS) for 1 h at room temperature (RT). For anti-ASPA and anti-BCAS1 immunostaining, brain sections were subjected to an antigen retrieval procedure prior to permeabilization by incubating brain sections in 10 mM citrate buffer (5 mM citric acid, 0.05% Tween 20, pH 6.0) at 95°C for 3–5 min, and then rinsed with PBS (5 min, three times) before the permeabilization step. After the blocking, sections were incubated in a blocking solution containing primary antibodies at 4°C overnight. The primary antibodies used in this study were rabbit anti- β-Amyloid (D54D2, Cell Signaling Technology Cat#8243; 1:500), mouse anti-β Amyloid (6E10, Covance Ca#SIG-39320; 1:500), rabbit anti-ASPA (GeneTex Cat#GTX113389; 1:500), rabbit anti-GFAP (Agilent, Cat# Z033429-2; 1:500), chicken anti-GFP (Aves Labs, Cat#GFP1020; 1:1,000), goat anti-GFP (Rockland, Cat# 600-101-215; 1:500), rabbit anti-Iba1 (Wako, Cat#019-19741; 1:500), mouse anti-MBP (Covance, Cat# SMI-99P; 1:500), rabbit anti-MBP (Cell Signaling Technology, Cat#78896; 1:500), guinea pig anti-NG2 (generated in Bergles’ Lab, Johns Hopkins University; 1:500), guinea pig anti-BCAS1 (Synaptic systems, Cat# 445004; 1:300), rabbit anti-NF200 (Sigma-Aldrich, Cat#N4142; 1:1,000), rabbit anti-Caspr (abcam, Cat#34151; 1:500), rabbit anti-Olig2 (Sigma Millipore, Cat# 387R-14; 1:500), and goat anti-Sox9 (R & D Systems, Cat# AF3075, 1:500). After the primary antibody incubation, those sections were rinsed with PBS (5 min, three times) and incubated with secondary antibodies and DAPI (1:1,000) in the blocking solution at RT for 2 h. Secondary antibodies used in this study were Alexa Fluor 488-, Cy3-, Cy5-, or Alexa Fluor 647- conjugated donkey IgG against chicken, goat, mouse, rabbit, or guinea pig (Jackson ImmunoResearch, 1:500). Sections were rinsed in PBS (for 5 min, three times) and then mounted onto slide glasses with a mounting medium Fluoromount-G (SouthernBiotech).

### Selection of Hippocampal Subregions for Cell Quantification

Four hippocampal subregions were selected for OL quantification: (1) stratum lacunosum-moleculare (SLM) of CA1, (2) the dentate gyrus (DG) hilus (CA4), (3) stratum lucidum (SL) and stratum radiatum (SR) of CA3, and (4) SR of CA1 (see Figure 2A). The rationale for this regional selection is related to the importance of major hippocampal circuits and their axonal projection. The SLM of CA1 includes axons of the direct perforant pathway originating from layer III of the entorhinal cortex (EC) (Suh et al., 2011). The DG, CA3, and SR + SO of CA1 constitute the indirect perforant pathway and represent relays of three synaptic connections. In the indirect perforant path, layer II neurons of the EC project to dendrites of DG granule cells in the DG molecular layer. The dentate granule cells project their axons to CA3 through CA4, the DG hilar region, forming mossy fibers. However, the CA4 and CA3 areas also include various types of interneurons (Markwardt et al., 2011; Pelkey et al., 2017). The CA3 pyramidal neuron axons project to SR of the CA1, which forms the Schaffer collateral pathway.

### Image Acquisition

Fluorescent images were captured using Axio-Imager M2, an epifluorescence microscope (Zeiss), and the Axiovision (7.0) software (Zeiss). Three to 4 sections were assessed per mouse, and at least three mice per group were used for analysis. Hippocampal subregions were determined based on DAPI-based nuclear staining patterns and *The Mouse Brain in Stereotaxic Coordinates* (Franklin and Paxinos, 2008). Confocal images were obtained with a laser scanning microscope TCS SP8 (Leica) and processed with LAS X software (Leica).

### Quantification and Statistical Analysis

Cell counting and hippocampal subregion outlining were manually performed using the ZEN software (Zeiss). The percentage of the area of Aβ plaques and mean pixel values of MBP^+^ or NF200^+^ were determined using Image J. Prism 9.0 (GraphPad) was used for the statistical analysis and graph drawing. Statistical significance was determined with a two-tailed unpaired Student’s *t*-test (for two-group comparisons) or one-way ANOVA with the Tukey *post-hoc* test (more than two-group comparisons) or one-way ANOVA with Dunnett’s test (for comparisons with a common control). When effects of age or AD-like conditions are compared across hippocampal subregions, two-way ANOVA and Sidak multiple comparison tests were performed. Error bars represent the standard error of the mean (SEM). “*n*” represents the number of mice used in each experiment, and three or four mice per group were used unless otherwise stated.

## Data Availability Statement

The original contributions presented in the study are included in the article/Supplementary Material, further inquiries can be directed to the corresponding author.

## Ethics Statement

The animal study was reviewed and approved by the Temple University IACUC.

## Author Contributions

LD performed immunostaining and quantitative analysis, interpreted results, and wrote the manuscript. EG-F sampled mice and performed confocal microscopy. IC sampled mice, performed immunostaining, and quantitative analysis. SK conceived, designed, and supervised the study, interpreted results, and wrote the manuscript. All authors contributed to the article and approved the submitted version.

## Conflict of Interest

The authors declare that the research was conducted in the absence of any commercial or financial relationships that could be construed as a potential conflict of interest.

## Publisher’s Note

All claims expressed in this article are solely those of the authors and do not necessarily represent those of their affiliated organizations, or those of the publisher, the editors and the reviewers. Any product that may be evaluated in this article, or claim that may be made by its manufacturer, is not guaranteed or endorsed by the publisher.
