# Supplementary figures and images for: Age and Alzheimer’s Disease-Related Oligodendrocyte Changes in Hippocampal Subregions
Source: Front Cell Neurosci. 2022 Apr 7;16:847097. doi: 10.3389/fncel.2022.847097 (PMC9023310; doi:10.3389/fncel.2022.847097)

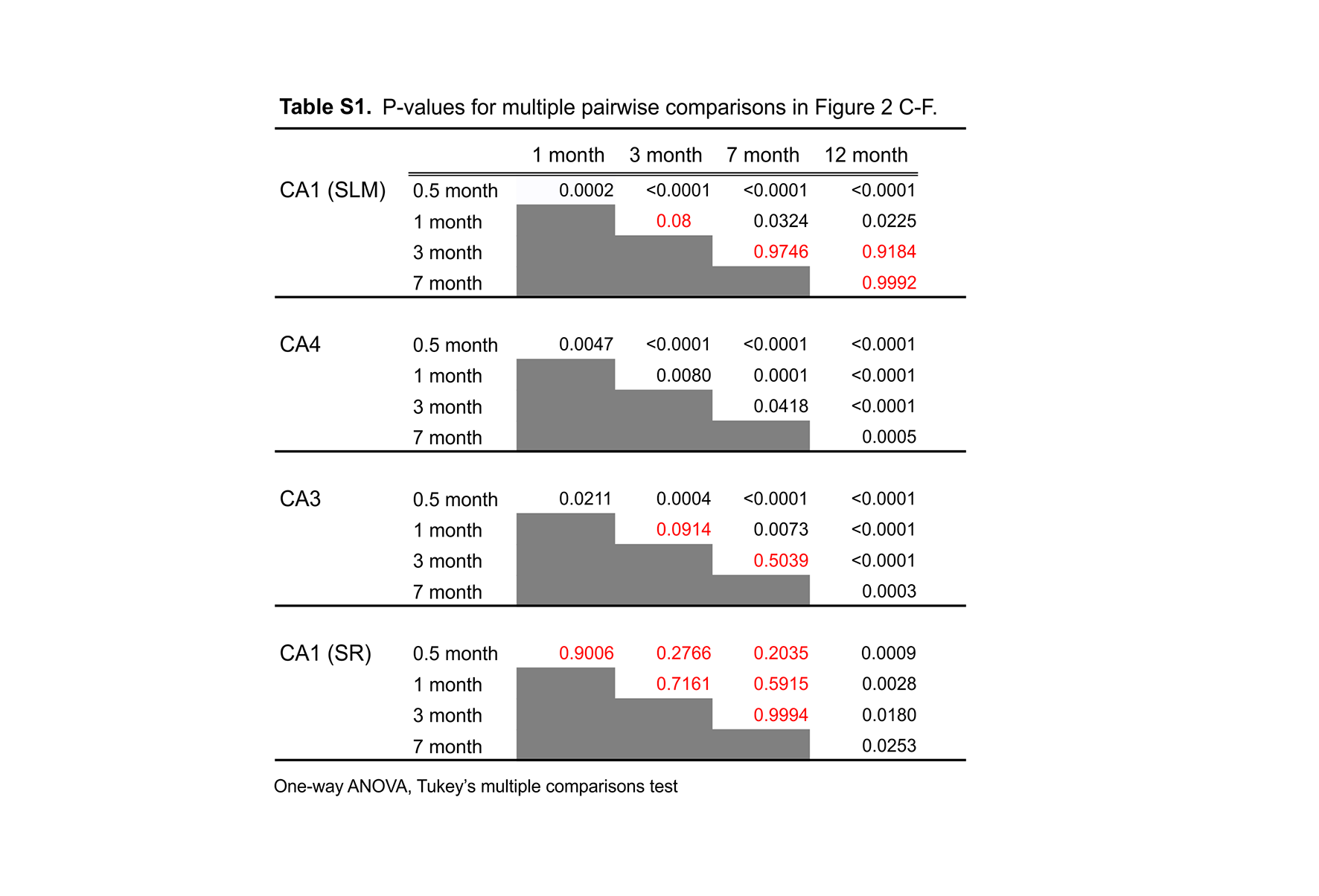

Supplement: Supplementary file 1 [file Image_1.TIF]
